# Platelet, Mean Platelet Volume and Platelet Distribution Width Levels Might Be a Promising Marker for the Prediction of Disease Severity, Mucosal Healing and Steroid Dependence in Patients With Ulcerative Colitis

**DOI:** 10.7759/cureus.33286

**Published:** 2023-01-02

**Authors:** Emre Gerçeker, Serkan Cerrah

**Affiliations:** 1 Gastroenterology, İzmir Medicana International Hospital, İzmir, TUR; 2 Gastroenterology and Hepatology, Erzurum Regional Training and Research Hospital, Erzurum, TUR

**Keywords:** ulcerative colitis, steroid dependence, platelet distribution width, platelet, mucosal healing, mean platelet volume

## Abstract

Background/aims: In this study, we aimed to determine the value of mean platelet (PLT) volume (MPV), PLT and PLT distribution width (PDW) levels as a marker in the prediction of mucosal healing (MH), steroid resistance (SR) and steroid dependence (SD) in newly diagnosed moderate and severely active patients with ulcerative colitis (UC), who did not receive medical treatment before.

Patients/methods: Two hundred forty-nine patients with severely or moderately active UC and 50 healthy subjects were enrolled in the study after retrospective analysis. Disease severity and MH of UC were determined according to the Mayo Score. According to the results of remission induction therapy, the patients were divided into two groups: Group 1; MH positive and Group 2; MH negative. UC patients with clinical remission (CR) but without MH were divided into two subgroups SD and non-SD during their follow-up. These two groups and subgroups were compared for variables.

Results: 42.6% of patients with UC had severe disease activation. 44.6% of patients with UC had pancolitis. After remission induction therapy, CR was observed in 84.3% of patients with UC. MH rate was 53.0%. SR rate was 15.7% and the SD rate was 16.1%. A strong positive correlation was observed between C reactive protein (CRP), PLT and Mayo score in the activation period (r=0.835 and p<0.001; r=0.883 and p<0.001; respectively). A strong negative correlation was observed between mean PLT volume (MPV), PDW levels and Mayo score (r=-0.905 and p<0.001; r=-0.805 and p<0.001; respectively). According to the receiver operating characteristic curve (ROC) analysis, PLT had a sensitivity of 42.4% and a specificity of 22.7% in the prediction of MH at a cut-off value of 266.5x10^3^/µL. MPV had a sensitivity of 83.5% and a specificity of 73.5% in the prediction of MH at a cut-off value of 8.05 fL. PDW had a sensitivity of 88.6% and a specificity of 84.5% in the prediction of MH at a cut-off value of 2.95 fL. PLT was determined with 92.5% sensitivity and 86.8% specificity in the prediction of SD at a cut-off value of 287.0x10^3^/µL. MPV had a sensitivity of 86.8% and a specificity of 67.5% in the prediction of SD at a cut-off value of 7.95 fL. PDW had a sensitivity of 73.7% and a specificity of 72.5% in the prediction of SD at a cut-off value of 12.55 fL.

Conclusions: There was a positive correlation between PLT levels and Mayo score, and a negative correlation between Mayo score and MPV or PDW levels. We think that PLT, MPV and PDW levels may be promising markers in the evaluation of disease activation/remission and severity. We believe that PLT, MPV and PDW levels will be determinative especially in the exclusion of SD, for UC patients with CR but without MH.

## Introduction

Ulcerative colitis (UC) is a chronic inflammatory disease that progresses with periods of chronic exacerbation and remission, especially involving the colon. Activation periods significantly impair the quality of life [1]. The primary goal of treatment is to improve the quality of life by achieving clinical remission (CR). Secondly, it is to prevent relapse by providing mucosal healing (MH). While oral and enema forms of mesalazine are sufficient in remission induction treatment in mild UC, steroids are the first choice in moderate to severe UC [2].

As it is known, steroid resistance (SR) or steroid dependence (SD) may develop in 20%-30% of patients with UC, although the response is obtained in most cases with steroids in the remission induction treatment of moderate to severe UC. [3]. While the cause of this resistance or dependence may sometimes be underlying viral or bacterial infections, sometimes any occasion cannot be identified. For induction of remission in case of primary unresponsiveness (development of SR) and maintenance of remission in SD, advanced treatments such as cyclosporine, anti-tumor necrosis factor alpha (anti-TNF α), anti-interleukin, and anti-integrin should be started [4-6].

Today, there is no marker that determines which cases will develop SR or SD. The decision of CR and MH are determined by clinical symptoms, endoscopic findings, fecal calprotectin and C reactive protein (CRP) levels. Although SR can be predicted by clinical, radiological and laboratory findings, a prediction cannot be made always due to the clinical and laboratory improvement in SD. As it is known, side effects occur in the long-term use of steroid therapy. In addition, failure to achieve remission induction leads to increased complications related to UC and even the need for colectomy. Therefore, early detection of failure of first-stage remission induction therapy is important. This has led clinicians to seek early markers [6,7].

It is known that mean platelet (PLT) volume (MPV), PLT, and PLT distribution width (PDW) levels change during the activation period of many chronic inflammatory diseases [8]. In clinical studies, especially in the active phase of UC, compared to both the normal population and patients with UC in the remission phase; It has been reported that there is an increase in PLT levels and a decrease in MPV and PDW levels [9-13]. Activation of PLT is also observed during the exacerbation period of UC. During the increased thrombopoiesis, the PLT level increases rapidly. However, PLT abnormalities and changes in megakaryopoiesis are caused by an inflammatory state on low MPV, PDW and reticulated PLT values during the course of UC [14]. It has been shown in some studies, that UC disease activation severity and prognosis were positively correlated with PLT and negatively correlated with MPV and PDW [9-13]. In our study, we planned to investigate the value of MPV, PLT and PDW levels as a marker for the prediction of MH and SD in newly diagnosed moderately and severely active patients with UC, who did not receive medical treatment before.

## Materials and methods

Study design and patients

The study was carried out in the Gastroenterology Department of Erzurum Regional Training and Research Hospital. Before starting the study, approval was obtained from the Clinical Research Ethics Committee of Erzurum Regional Training and Research Hospital (IRB approval number: 2022/18-177). Two hundred and forty-nine (n=249) patients over the age of 18 who were admitted to our clinic with newly diagnosed moderately or severely active UC according to Mayo score between January 2018 and July 2022, were included in the study. The diagnosis of UC was established by using clinical, endoscopic, histologic, and radiologic parameters. Cases who applied for UC activation and had a previous diagnosis of UC were not included in the study. Fifty cases who were examined in the gastroenterology clinic of our hospital due to dyspeptic symptoms and diagnosed only with irritable bowel syndrome were included in the study as the control group. Patients with a previous history of malignancy, chronic rheumatic disease (connective tissue disease, etc.), and hematological disease were not included in the study. Patients who were using anticoagulant or antiaggregant drugs at the time of diagnosis were not included in the study.

In all patients, age, gender, diagnosis of other concomitant systemic diseases, the extent of disease involvement, duration of disease, disease activation severity, extra-intestinal involvement, and medical treatments for the disease were retrospectively scanned and recorded. Complete blood count (CBC), routine biochemical (liver transaminase, albumin), and imaging tests such as esophagogastroduodenoscopy, colonoscopy, abdominal computed tomography, and magnetic resonance imaging performed at the time of admission were retrospectively scanned and recorded. The severity of the disease was determined according to the Mayo scoring system. A Mayo score between 6 and 10 was considered moderate and a Mayo score of 11-12 was considered a severe disease. The cases were excluded from the study if they were positive for cytomegalovirus, tuberculosis, Entamoeba histolytica, and Clostridium difficile.

Clinical follow-up and medical treatment

After the initial evaluation, medical treatment was initiated for all patients according to the guidelines of the European Crohn's and Colitis Organization [2]. 3-4 g/day mesalazine and 1 mg/kg/day methyl-prednisolone were started as remission induction therapy for all newly diagnosed patients with moderate to severe UC. Severe cases were treated and followed up in a hospital setting. Moderate cases were followed up in an outpatient clinic once a week for the first month and every 4 weeks thereafter. All patients were re-evaluated clinically, laboratory, and endoscopically during the follow-up period. Clinical outcomes such as CR, MH, and the development of SR and SD during the follow-up period of all cases were recorded. Cases that did not have abdominal pain, rectal bleeding, diarrhea, and also had decreased CRP levels in laboratory tests were accepted as in CR [2]. After the start of the treatment, MH was evaluated by colonoscopy at the earliest four weeks, at the latest 12 weeks. Cases with a Mayo score ≤ 2 were considered as MH [2].

Steroid resistance/dependence

SR was defined as an active disease despite methyl-prednisolone up to 0.75-1.00 mg/kg per day over a period of at least four weeks. Patients with SD were defined as those who are either unable to reduce steroids below the equivalent of methyl-prednisolone 10 mg/day within three months of initiation without recurrent active disease or who have a relapse within three months after the end of the therapy [2].

Patients' groups

According to the results of remission induction therapy, the patients were divided into two groups: Group 1; MH positive, and Group 2; MH negative. UC patients with CR but without MH development were divided into two subgroups SD and non-SD (n=38) during their follow-up. These two groups and subgroups were compared for variables.

Statistics

Parameters were made by using the “Statistical Package for the Social Sciences (SPSS) 22 for Windows” statistics program. Categorical (nominal) values were expressed as a percentage (%) and compared with the chi-square test (2). Continuous numerical (quantitative) values were expressed as mean ± standard deviation (SD). Pearson chi-square (χ2) test was used in the comparison analysis for categorical variables and the Mann-Whitney U test was used in the comparison analysis for continuous variables between the groups and subgroups. Bivariate Spearman correlation analysis was used to determine the correlation between disease MPV, PDW, CRP, PLT levels, and disease activation severity (Mayo score). Receiver operating characteristic (ROC) curve analysis was performed to calculate the predictive value of MPV, PDW, CRP, and PLT levels in the determination of MH or SD. According to the ROC analysis, the area under the ROC curve (AUC) and cut-off values, sensitivity, and specificity of these cut-off values were presented. If p < 0.05 was determined as statistically significant.

## Results

Two hundred forty-nine newly diagnosed UC patients with moderate to severe activity and 50 control cases were included in this study. Demographic characteristics of patients with UC and control cases were summarized in Table 1. The distribution of age, gender, liver function tests, albumin levels, and spleen diameter variables were similar in both groups. WBC, PLT, and CRP levels were significantly higher in the UC group than in the control group (8.35x10^3^/µL vs 7.15x10^3^/µL and p<0.001; 317.90x10^3^/µL vs 276.70x10^3^/µL and p<0.001; 59.38 mg/L vs 3.10 mg/L and p<0.001, respectively). MPV, PDW, and hemoglobin levels were found to be lower (7.80 fL vs 8.83 fL and p<0.001; 12.17 fL vs 13.32 fL and p<0.001; 11.93 g/dL vs 12.63 g/dL and p=0.008, respectively).

**Table 1:** Demographic characteristics of patients with UC and control cases ALT: Alanine transaminase, AST: Aspartate transaminase, CRP: C-reactive protein, MPV: Mean platelet volume, PDW; Platelet distribution width, PLT: Platelet, WBC: White blood cell, UC: Ulcerative colitis

Variable	UC (n=249)	Control (n=50)	P-value
Gender (Female)	55.8%	62%	0.439
Age (Year)	40.59 ± 11.40	39.98 ± 9.92	0.698
CRP (mg/L)	59.83 ± 28.35	3.10 ± 1.50	<0.001
WBC (x10^3^ µL)	8.35 ± 3.25	7.15 ± 1.62	<0.001
Hemoglobin (gr/dL)	11.93 ± 2.06	12,63 ± 1.59	0.008
PLT (x10^3^ µL)	317.90 ± 30.24	276.70 ± 41.47	<0.001
MPV (fL)	7.80 ± 0.19	8.83 ± 0.50	<0.001
PDW (fL)	12.17 ± 0.77	13.32 ± 0.36	<0.001
AST (IU/L)	24,71 ± 13.80	21.74 ± 5.66	0.108
ALT (IU/L)	19.59 ± 11.41	18.80 ± 9.31	0.547
Albumin (g/dL)	3.58 ± 0.59	3.54 ± 0.57	0.812
Spleen Diameter (mm)	93.15 ± 5.47	93.22 ± 5.48	0.880

42.6% of patients with UC had severely disease activation. 44.6% of patients with UC had pancolitis involvement. After remission induction therapy, CR was observed in 84.3% of patients with UC. MH rate was 53.0%. It was determined that 15.7% of UC patients developed SR and 16.1% developed SD.

The comparison of inflammatory markers between the MH group and the non-MH group was summarized in Table 2. When the MH positive group was compared with MH negative group, no difference was found for age, gender, disease severity, initial mayo score, and colonic involvement patterns. WBC, hemoglobin, PLT, MPV, PDW, CRP, liver transaminase, albumin, and spleen diameter values at the time of diagnosis were similar in both groups. In the MH-positive group, significantly higher MPV and PDW levels were observed in the remission period compared to the non-MH group (8.30 fL vs 7.93 fL and p<0.001; 13.21 fL vs 12.40 fL and p<0.001, respectively). In the MH-positive group, CRP and PLT levels were found to be lower during the remission period (2.96 mg/L vs 30.90 mg/L and p<0.001; 263.64x10^3^/µL vs 301.03x10^3^/µL and p<0.001, respectively).

**Table 2:** The comparison of inflammatory markers between the mucosal remission and non-mucosal remission groups ALT: Alanine transaminase, AST: Aspartate transaminase, CRP1: C-reactive protein level before treatment, CRP2: C-reactive protein level before treatment, Mayo Score1: Mayo score before treatment, Mayo Score2: Mayo score after treatment, MPV1: Mean platelet volume level before treatment, MPV2: Mean platelet volume level after treatment, PDW1: Platelet distribution width level before treatment PDW2: Platelet distribution width level after treatment, PLT1: Platelet level before treatment PLT2: Platelet level after treatment, WBC: White blood cell, UC: Ulcerative colitis

Variable	Remission (n=132)	Non-remission (n=117)	P-value
Gender (Female)	54.5%	57.3%	0.702
Age (Year)	39.61 ± 11.08	41.71 ± 11.71	0.147
Severe Colitis	40.9%	44.4%	0.609
Pancolitis	46.2%	42.7%	0.611
CRP^1^ (mg/L)	58.07 ± 32.04	61.82 ± 23.48	0.298
CRP^2^ (mg/L)	2.96 ± 1.60	30.90 ± 28.14	<0.001
Mayo Score^1^	8.83 ± 1.91	9.08 ± 1.89	0.313
Mayo Score^2^	6.99 ± 1.84	0.70 ± 0.75	<0.001
PLT^1^ (x10^3^ µL)	316.51 ± 29.26	319.48 ± 31.37	0.440
PLT^2^ (x10^3^ µL)	263.64 ± 17.80	301.03 ± 35.06	<0.001
MPV^1^ (fL)	7.81 ± 0.19	7.79 ± 0.21	0.963
MPV^2^ (fL)	8.30 ± 0.24	7.93 ± 0.16	<0.001
PDW^1^ (fL)	12.22 ± 0.89	12.12 ± 0.61	0.331
PDW^2^ (fL)	13.21 ± 0.44	12.40 ± 0.46	<0.001
WBC (x10^3^ µL)	8.33 ± 3.18	8.80 ± 3.58	0,276
Hemoglobin (gr/dL)	11.83 ± 2.12	11.43 ± 2.10	0.135
AST (IU/L)	26.0 ± 10.30	23.18 ± 16.83	0.109
ALT (IU/L)	19.65 ± 9.80	19.51 ±13.03	0.924
Albumin (g/dL)	3.61 ± 0.63	3.54 ± 0.53	0.392
Spleen Diameter (mm)	93.20 ± 5.48	93.10 ± 5.49	0.892

The relationship between the Mayo score and serum inflammatory parameters was summarized in Tables 3, 4. A strong positive correlation was observed between CRP, PLT, and Mayo score at the time of first diagnosis (r=0.835 and p<0.001; r=0.883 and p<0.001; respectively). A strong negative correlation was observed between MPV, PDW levels and Mayo score (r=-0.905 and p<0.001; r=-0.805 and p<0.001, respectively). A strong positive correlation was observed between CRP, PLT, and Mayo scores calculated in the post-treatment evaluations (r=0.772 and p<0.001; r=0.714 and p<0.001, respectively). A strong negative correlation was observed between MPV, PDW levels and Mayo score (r=-0.764 and p<0.001; r=-0.754 and p<0.001, respectively).

**Table 3:** The relationship between Mayo score and serum inflammatory parameters at the time of diagnosis CRP: C-reactive protein level before treatment, MPV: Mean platelet level before treatment, PDW: Platelet distribution width level before treatment, PLT: Platelet level before treatment, WBC: White blood cell before treatment

Mayo Score	r	p-value
CRP (mg(dL)	0.835	<0.001
PLT (x10^3^ µL)	0.883	<0.001
MPV (fL)	-0.905	<0.001
PDW (fL)	-0.805	<0.001
Hemoglobin (gr/dL)	-0.488	<0.001
WBC (x10^3^ µL)	0.299	<0.001

**Table 4:** The relationship between Mayo score and serum inflammatory parameters in post-treatment evaluation CRP: C-reactive protein level after treatment, MPV: Mean platelet level after treatment, PDW: Platelet distribution width level after treatment, PLT: Platelet level after treatment

Mayo Score	r	P-value
CRP (mg(dL)	0.772	<0.001
PLT (x10^3^ µL)	0.714	<0.001
MPV (fL)	-0.764	<0.001
PDW (fL)	-0.754	<0.001

ROC analysis of inflammatory markers for predictive value to differentiate MH was summarized in Table 5. According to the ROC analysis, CRP were determined with 88.0% sensitivity and 81.9% specificity in the prediction of MH at a cut-off value of 5.05 mg/L. PLT had a sensitivity of 42.4% and a specificity of 22.7% in the prediction of MH at a cut-off value of 266.5x103/µL. MPV had a sensitivity of 83.5% and a specificity of 73.5% in the prediction of MH at a cut-off value of 8.05 fL. PDW had a sensitivity of 88.6% and a specificity of 84.5% in the prediction of MH at a cut-off value of 12.95 fL.

**Table 5:** ROC analysis of inflammatory markers for predictive value to differentiate mucosal healing CRP: C-reactive protein level after treatment, MPV: Mean platelet level after treatment, PDW: Platelet distribution width level after treatment, PLT: Platelet level after treatment

Variable	AUC (95% CI)	P-value	Cut-off	Sensitivity	Specificity
CRP (mg(dL)	0.951 (0.924-0.979)	<0.001	<5.05	88.0%	81.1%
PLT (x10^3^ µL)	0.815 (0.757-0.873)	<0.001	>266.5	42.4%	22.2%
MPV (fL)	0.902 (0.866-0.938)	<0.001	>8.05	83.5%	73.4%
PDW (fL)	0.917 (0.879-0.954)	<0.001	>12.95	88.6%	84.5%

UC patients with CR but without MH development (n=78) were divided into two subgroups as SD (n=40) and non-SD (n=38) during their follow-up. These two subgroups were compared for variables. The results are summarized in Table 6. When the subgroup with SD was compared with the subgroup without SD, no difference was found for age, gender, disease severity, initial mayo score and colonic involvement patterns. WBC, hemoglobin, platelet, MPV, CRP, liver transaminase, albumin, and spleen diameter values at the time of diagnosis were similar in both groups. Although CRP and Mayo score levels were similar after treatment, in the non-SD group significantly higher MPV and PDW levels were observed in the remission period compared to the SD subgroup (8.07 fL vs 7.89 fL and p<0.001; 12.67 fL vs 12.41 fL and p<0.001; respectively). In the subgroup with non-SD, PLT levels were found to be lower during the remission period (262.82x10^3^/µL vs 308.55x10^3^/µL and p<0.001, respectively).

**Table 6:** Comparison of the variables between SD positive and negative patients with UC who are MH negative but CR positive ALT: Alanine transaminase, AST: Aspartate transaminase, CRP1: C-reactive protein level before treatment, CRP2: C-reactive protein level after treatment, Mayo Score1: Mayo score before treatment, Mayo Score2: Mayo score after treatment, MPV1: Mean platelet volume level before treatment, MPV2: Mean platelet volume level after treatment, PDW1: Platelet distribution width level before treatment PDW2: Platelet distribution width level after treatment, PLT1: Platelet level before treatment PLT2: Platelet level after treatment, SD: Steroid dependence, WBC: White blood cell, UC: Ulcerative colitis

Variable	SD (n=40)	Non-SD (n=38)	P-value
Gender (Female)	57.5%	57.9%	0.972
Age (Year)	42.45 ± 11.14	40.5 ± 12.19	0.463
Severe Colitis	47.5%	44.7%	0.824
Pancolitis	42.5%	44.7%	0.843
CRP^1^ (mg/L)	59.55 ± 24.56	61.16 ± 24.57	0.773
CRP^2^ (mg/L)	12.53 ± 5.97	11.90 ± 7.44	0.685
Mayo Score^1^	9.28 ± 2.03	9.05 ± 1.86	0.615
Mayo Score^2^	6.10 ± 0.98	5.89 ± 1.16	0.402
PLT^1^ (x10^3^ µL)	319.15 ± 33.23	312.13 ± 30.13	0.332
PLT^2^ (x10^3^ µL)	308.55 ± 16.47	262.82 ± 23.10	<0.001
MPV^1^ (fL)	7.79 ± 0.18	7.77 ± 0.17	0.643
MPV^2^ (fL)	7.89 ± 0.11	8.07 ± 0.11	<0.001
PDW^1^ (fL)	12.03 ± 0.67	12.15 ± 0.63	0.417
PDW^2^ (fL)	12.41 ± 0.27	12.67 ± 0.36	<0.001
WBC (10^3^ µL)	8.48 ± 3.42	8.93 ± 3.72	0.577
Hemoglobin (gr/dL)	12.09 ± 1.98	11.59 ± 2.26	0.296
AST (IU/L)	20.57 ± 5.39	22.44 ± 9.39	0.279
ALT (IU/L)	17.65 ± 8.06	17.97 ± 9.62	0.872
Albumin (gr/dL)	3.46 ± 0.48	3.59 ± 0.54	0.260
Spleen Diameter (mm)	93.68 ± 5.77	92.53 ± 5.57	0.374

ROC analysis of inflammatory markers for predictive value to differentiate SD were summarized in Table 7. According to the ROC analysis, PLT was determined with 92.5% sensitivity and 86.8% specificity in the prediction of SD at a cut-off value of 287.0x10^3^/µL. MPV had a sensitivity of 86.8% and a specificity of 67.5% in the prediction SD at a cut-off value of 7.95 fL. PDW had a sensitivity of 73.7% and a specificity of 72.5% in the prediction SD at a cut-off value of 12.55 fL.

**Table 7:** ROC analysis of inflammatory markers for predictive value to differentiate steroid dependence CRP: C-reactive protein level after treatment, MPV: Mean platelet level after treatment, PDW: Platelet distribution width level after treatment, PLT: Platelet level after treatment

Variable	AUC (95% CI)	P Value	Cut-off	Sensitivity	Specificity
CRP (mg(dL)	0.550 (0.420-0.680)	0.447	11.15	62.5%	50%
PLT (10^3^ µL)	0.941 (0.884-0.997)	<0.001	287.0	92.5%	86.8%
MPV (fL)	0.869 (0.789-0.949)	<0.001	<7.95	86.8%	67.5%
PDW (fL)	0.788 (0.685-0.891)	<0.001	<12.55	73.7%	72.5%

## Discussion

In addition to its primary hemeostatic functions, PLT is also involved in the pathogenesis of chronic inflammatory diseases, especially inflammatory bowel disease [15]. Activation of platelets plays a role in determining and managing mucosal inflammation in the active phase of UC by affecting cytokine levels, such as IL-1, beta-tromboglobulin, platelet-derived growth factor, platelet activation factor, platelets factor 4 [10,16]. Finally, it is known that MPV and PDW levels show influence during exacerbations of chronic inflammation [8-13]. In the present study, we aimed to determine the predictive value of PLT, MPV, and PDW levels in detecting disease activation, disease severity, and MH in patients with UC. Similar to the literature [8], in the current study, PLT levels were found to be significantly higher, and MPV and PDW levels were lower in patients with active UC compared to the control group or group of UC patients with MH.

In some studies, it has been reported that there is a positive correlation between disease severity and PLT level, and a negative correlation between MPV and PDW levels [9-13]. Yüksel et al. [9] reported that MPV level was lower in UC patients than in the normal population, and it was lower in active patients than in remission patients. In the same study, they stated that there was a negative correlation between MPV level and disease severity. The overall accuracy of MPV for the determination of active UC was 71% (with a sensitivity 67%, and specificity of 73%) in the aforementioned study. Chen et al. [12] reported that the level of MPV and PLT changed during the UC exacerbation period. They found that disease severity and MPV level showed a negative correlation, and PLT level showed a positive correlation. They pointed out that both parameters can be used as a marker for disease activity indicators. Öztürk et al. [10] stated that MPV and PDW levels in the active phase were lower than those in the remission phase and control group. They also reported that both parameters showed a negative correlation with disease severity. They predicted that it can be used to differentiate the active and remission phases of the disease. They reported that the active phase of the disease could be detected with MPV cut-off level <8.2 fL (60.0% Sensitivity and 77.5% Specificity) and PDW cut-off level <16.6 fL (60.0% Sensitivity and 77.5% and Specificity). Cifci et al. [11] found that UC disease severity was positively correlated with CRP, and PLT levels and negatively correlated with MPV. They reported that PLT level was predictive in the detection of MH (Cut-off value <278.6x10^3^/µL; Sensitivity and 72.2% Specificity 46.2%), but MPV levels were not statistically significant. According to the meta-analysis of Bambo et al. [17], MPV levels were lower in UC compared to the control. The decreased MPV levels were associated with the progression and severity of UC activity. MPV levels can provide diagnostic and prognostic information in UC disease courses.

In our study, a positive correlation was found between PLT levels and the initial Mayo score, which is an indicator of disease severity. It detected a negative correlation between the initial Mayo score and MPV, and PDW levels, in line with the literature. In addition, a decrease in PLT levels was observed in patients with MH after treatment, positively correlated with the Mayo score. Likewise, an increase in MPV and PDW values was observed negatively correlated with the Mayo score in which MH was detected in the post-treatment evaluation. In cases where MH could not be achieved, MPV, PDW, and PLT levels were similar before and after treatment. In line with these findings, we think that PLT, MPV, and PDW levels may be promising markers in the evaluation of disease activation/remission and severity. According to the ROC analysis, PLT had a sensitivity of 42.4% and a specificity of 22.7% in the prediction of MH at a cut-off value of 266.5x10^3^/µL. MPV had a sensitivity of 83.5% and a specificity of 73.5% in the prediction of MH at a cut-off value of 8.05 fL. PDW had a sensitivity of 88.6% and a specificity of 84.5% in the prediction MH at a cut-off value of 12.95 fL.

Although CR is the primary goal for patients with UC to improve their quality of life, MH is the most important target to reduce clinical relapses, ensure long-term remission, and prevent complications [18-20]. In some of the patients with UC in whom CR is observed but MH cannot be achieved at first control colonoscopy examination, MH and a long-term exacerbation-free period can be achieved by continuing the current remission induction therapy. On the other hand, in some of the patients with UC, steroid treatment cannot be stopped/reduced and clinical exacerbations may develop in the early period [2-6,21]. This patient with UC is defined as the patient group with SD [2,6,21]. Separating these two groups is important in the transition to second-line treatment, such as anti-TNF α, anti-interleukin, and anti-integrin [2,4-6,21]. Currently, there is no early marker for detecting SD. The present study is the first to show the importance of MPV, PDW, and PLT levels as a predictive early marker for SD in cases with CR but not MH. In our study, we observed that in the subgroup of UC patients with CR without MH, high PLT values regressed in UC patients without SD, while low MPV and PDW values increased. On the contrary, we observed that there was no significant change in PLT, MPV, and PDW values in UC patients with SD. According to the ROC analysis, we determined the cut-off values at a good sensitivity and specificity ratio. PLT was determined with 92.5% sensitivity and 86.8% specificity in the prediction of SD at a cut-off value of 287.0 x10^3^/µL. MPV had a sensitivity of 86.8% and a specificity of 67.5% in the prediction of SD at a cut-off value of 7.95 fL. PDW had a sensitivity of 73.7% and a specificity of 72.5% in the prediction SD at a cut-off value of 12.55 fL. We believe that these obtained values will be determinative, especially in the exclusion of SD.

Strengths of our work

It is possible to reach detailed patient data in the periods when the cases applied with activation and in the post-treatment follow-ups, and to reach a sufficient number of patients. Although this is a retrospective study, it enabled us to make an adequate and detailed analysis. A positive correlation between the Mayo score and PLT, a negative correlation between the Mayo score and MPV, and PDW could be determined by analyzing. In addition, the cut-off values of PLT, MPV, and PDW could be detected for the detection of MH or SD.

When we evaluate the limitations of our study, the biggest inadequacy is that it is a retrospective study. We believe that in a prospective study, the correlation between PLT, MPV, PDW, and Mayo scores could be determined more clearly. In addition, the optimal cut-off values of PLT, MPV, and PDW for the detection of MH or SD could be more clearly in a prospective study.

## Conclusions

PLT levels were found to be significantly higher, and MPV and PDW levels were found to be lower in patients with active UC compared to the control group. There was a positive correlation between PLT levels and the initial Mayo score, which is an indicator of disease severity. It detected a negative correlation between the initial Mayo score with MPV and PDW levels, In the line with these findings, we think that PLT, MPV, and PDW levels may be promising markers in the evaluation of disease activation/remission and severity. We believe that PLT, MPV, and PDW levels will be determinative especially in the exclusion of SD, for UC patients with CR but without MH.
